# Knee Joint Biomechanical Gait Data Classification for Knee Pathology Assessment: A Literature Review

**DOI:** 10.1155/2019/7472039

**Published:** 2019-05-14

**Authors:** Mariem Abid, Neila Mezghani, Amar Mitiche

**Affiliations:** ^1^Centre de Recherche LICEF, TELUQ University, Montreal, Canada; ^2^Laboratoire de Recherche en Imagerie et Orthopédie (LIO), Centre de Recherche du CHUM, Montreal, Canada; ^3^Institut National de la Recherche Scientifique (INRS), Centre Énergie, Matériaux et Télécommunications, Montreal, Canada

## Abstract

**Background:**

The purpose of this study is to review the current literature on knee joint biomechanical gait data analysis for knee pathology classification. The review is prefaced by a presentation of the prerequisite knee joint biomechanics background and a description of biomechanical gait pattern recognition as a diagnostic tool. It is postfaced by discussions that highlight the current research findings and future directions.

**Methods:**

The review is based on a literature search in PubMed, IEEE Xplore, Science Direct, and Google Scholar on April 2019. Inclusion criteria admitted articles, written in either English or French, on knee joint biomechanical gait data classification in general. We recorded the relevant information pertaining to the investigated knee joint pathologies, the participants' attributes, data acquisition, feature extraction, and selection used to represent the data, as well as the classification algorithms and validation of the results.

**Results:**

Thirty-one studies met the inclusion criteria for review.

**Conclusions:**

The review reveals that the importance of medical applications of knee joint biomechanical gait data classification and recent progress in data acquisition technology are fostering intense interest in the subject and giving a strong impetus to research. The review also reveals that biomechanical data during locomotion carry essential information on knee joint conditions to infer an early diagnosis. This survey paper can serve as a useful informative reference for research on the subject.

## 1. Introduction

Classification of biomechanical gait patterns is a useful, promising diagnostic method to assess conditions such as knee joint injuries and pathologies. A thorough knowledge of the anatomy and biomechanics of the knee joint is essential to properly diagnose and treat such conditions [1]. We start with a brief introduction to knee joint anatomy [2] and biomechanics [3, 4].

The knee consists of two joints of three bones. The joints are the tibiofemoral joint (with medial and lateral compartments) and the patellofemoral joint. The bones are the femur superiorly, the tibia inferiorly, and the patella anteriorly. To maintain stability, the knee joint relies heavily on muscles and soft tissue structures, such as the cartilage, ligaments, and tendons.

**Figure 1:**
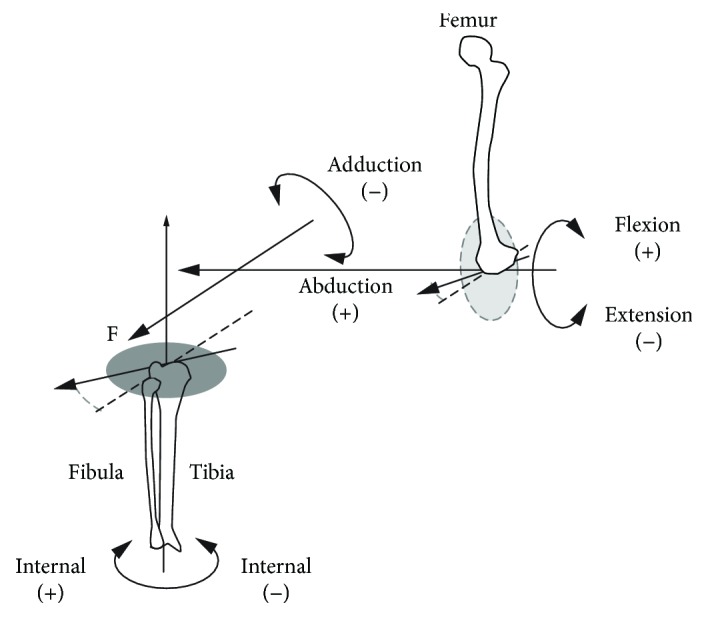
The knee joint coordinate system as defined by Grood and Suntay.

The knee joint function is to allow movements with six degrees of freedom: three rotational components about the axes of a coordinate system and three translational components along these. Usually, the Cartesian coordinate system is the reference system in biomechanics [5]. It consists of three axes: anterior-posterior, medial-lateral, and longitudinal, and the corresponding three planes are the frontal, the sagittal, and the transverse. The frontal plane divides the body into the front and back parts. The sagittal plane divides it into the left and right halves. Finally, the transverse plane divides the body into the top and bottom parts. The translations and rotations in the knee joint coordinate system have been described given Cartesian coordinate systems embedded in the tibia and femur [6]. In the knee joint coordinate system, flexion-extension occurs about the femoral axis, internal-external rotation occurs about the tibial axis, and abduction-adduction about an axis that is perpendicular to the femoral and tibial axes (as represented in Figure 1). The lateral-medial, distal-proximal, and posterior-anterior translations occur along each of the three coordinate axes, respectively.

Biomechanics is “the study of the movement of living things using the science of mechanics” [7], which is “a branch of physics that is concerned with the description of motion and how forces create motion” [5]. Biomechanics has already demonstrated the potential to be a useful tool in orthopaedics [8, 9]. The Chan et al.'s orthopaedic sport biomechanics paradigm [10] established the threefold role of biomechanics in (1) injury prevention, (2) immediate evaluation of treatment, and (3) long-term outcome evaluation. Labbe et al. [11] suggested adding two other roles to this paradigm, namely, (1) evaluating the impact of an injury on knee joint function and (2) assisting in diagnosis.

Biomechanical gait analysis studies involve recording a number of biomechanical variables including Electromyography (EMG) data, spatiotemporal parameters, kinematics, and kinetics [12]. EMG data corresponds to the electrical signals generated by muscular contraction. Spatiotemporal parameters include step and stride length, speed of movement, cadence, and single limb support (SLS). Kinematics is the study of movements without reference to the forces that cause motion, whereas kinetics is the study of forces that cause motion. Ground Reaction Forces (GRFs), joint moments, and joint powers are parts of the kinetic data.

The scientific literature relating to “gait analysis” in its most general meaning is abundant [13]. In human gait analysis, the two main topics of general interest are gait identification [14, 15] and gait analysis for clinical applications [16, 17]. Gait identification refers to the recognition of a person from their gait and is generally used as a biometric identifier. It is out of scope of the present review. This review addresses specifically biomechanical gait analysis for clinical applications, more precisely on the evaluation of the knee joint movement for a better understanding and diagnosis of knee joint injuries and pathologies. Examples of knee joint injuries and pathologies that can affect gait include ligament injury, meniscal tear, osteoarthritis (OA), patellofemoral syndrome, iliotibial band syndrome, bursitis, Baker's cyst, and tendinitis. Several studies of OA have recognized the importance of biomechanical gait analysis in the pathogenesis of the knee joint problems [18, 19]. The motivation for evaluating the knee joint movement is twofold. First, knee joints are the most commonly injured joints. There has been a report of 17,397 patients suffering 19,530 sports injuries in a 10-year span [20]. It showed that 6,434 (37%) had 7,769 injuries (39.8%) related to the knee joint. Second, common diagnostic methods of the knee joint problems, such as clinical assessment (medical history and physical examinations), roentgenography, magnetic resonance imaging, computed tomography, X-ray imaging, and arthroscopy, do not provide objective information on the functional aspects of the knee joint. For this reason, biomechanical assessment has become important for knee joint problem diagnosis; it provides quantitative information about the structure and motion of the knee joint to complement the common orthopaedic physical evaluation for more accurate diagnosis [21].

Knee joint movement data are first collected, using some measurement device, and characteristic features are extracted/selected for analysis. Biomechanical gait data analysis in clinical decision-making presents several difficulties. First, one is faced with large amounts of highly complex, multidimensional, highly variable, and time-dependent data [22]. Most of the data appear as temporal waveforms, i.e., time series. Second, reducing the amount of data and selecting its key features are a crucial step that can influence the results of the subsequent pattern classification. Time series parametrization and data transformation are two common ways to extract and select features to describe the data [23]. Finally, analysis involves a quantitative comparison of time series, a problem which may require complex processes to achieve stable and accurate results [24]. Chau achieved a two-part critical review of analytical techniques for gait data analysis [22, 25], and Ferber et al. investigated data science techniques [26]. In a nutshell, quantitative analysis methods of knee joint biomechanical gait data include statistical and Machine Learning (ML) techniques. A number of studies have addressed gait pattern classification, but the literature dealing specifically with knee joint biomechanical gait data classification is scarce.

**Figure 2:**
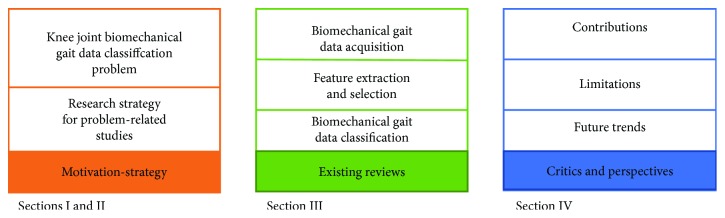
Roadmap of this survey.

Here following is a literature review and critical evaluation of the published literature on knee joint biomechanical gait data classification for computer-aided systems. We addressed the three major issues of knee joint biomechanical gait data classification we mentioned earlier, namely, (1) data acquisition, (2) feature extraction and selection, and (3) classification. The remainder of this paper, as the roadmap given in Figure 2 shows, is organized as follows: Section 2 describes the search strategy we adopted for the literature review on knee joint biomechanical gait data classification.  Section 3 provides a survey of pattern classification techniques for knee joint biomechanical gait data. Contributions and limitations of these studies as well as directions for future research are presented in Section 4.

## 2. Methods

We conducted a literature search on April 2019 using four electronic databases (PubMed, IEEE Xplore, Science Direct, and Google Scholar). We performed the search strategy using the following keywords (identical for all databases): “knee”, “gait”, and “classification”. 1,211 studies on knee joint biomechanical gait data classification have been selected. We also searched other reliable online articles such as thesis and book chapters for potentially eligible studies. In addition, review of all references cited by the selected articles and more insight into other relevant authors' reports yielded an additional 22 articles for possible inclusion.

**Figure 3:**
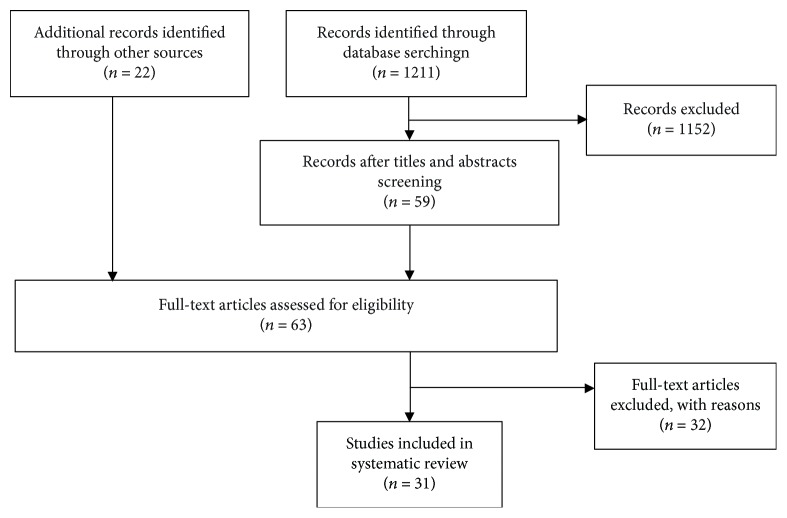
Flow of article inclusion/exclusion throughout the review process.

After evaluation of titles and abstracts, 59 articles were identified for possible inclusion. Removal of duplicates left 63 potential reports, from which 32 articles were excluded after full-text screening, as they did not meet the following inclusion/exclusion criteria. Articles included in the review were those published in peer-reviewed journals and conference proceedings and were written in either English or French. In addition, only studies conducted on the knee joint and where pattern recognition methods were performed on knee joint biomechanical data were eligible for inclusion. Kinematic, kinetic, spatiotemporal, and EMG parameters were biomechanical variables of interest. Studies relating to ankle, hip, limb, or foot and studies dealing with biometrics, inertial sensors, imaging, cerebral palsy, and Parkinson's disease were excluded. The search process is demonstrated using the flow diagram shown in Figure 3. All selected abstracts and full texts were indexed in Zotero software for subsequent analysis. As a result, we retained 31 full-text reports for full review, from which brought out information on the study participants, the data parameters (kinematic, kinetic, spatiotemporal, and EMG), and the research conclusions and contributions.

Next, we describe the full review results in terms of the basic constituent step of analysis as mentioned earlier, namely, (1) data acquisition, (2) feature extraction and selection, and (3) classification.

## 3. Results

We divided the 31 selected articles, which fulfilled the literature search inclusion criteria, into two distinct groups, according to data representation and classification techniques. We discuss briefly these categories in the following subsections, and a comparison table (Table 1) is given that inform on data acquisition techniques and accuracies. Note that we organized the reviews by relationship and group, so that works using similar methods are grouped together, and introduced in the chronological order of the publishing date.

### 3.1. Biomechanical Data Acquisition

Knee joint biomechanical gait data are collected during a walk session using recording equipment and software. Current data acquisition methods have been reviewed in [27]. In brief, a subject walks on a force platform that records the GRFs. Markers, active or passive, are generally fixed onto the human body segments and viewed by a motion capture system that records their three-dimensional (3D) trajectories. In a common setup, cameras collect data points representing the 3D coordinates of each marker during a treadmill session. These data (or trajectories) are transformed using rigid-body kinematics into knee joint angles, which describe the relative motion between knee segments over time, such as the angular displacement of the tibia with respect to the femur [28]. Joint angles combined with GRFs and inverse dynamic relations are then used to calculate joint moments in the three anatomical planes. In addition to temporal changes of joint angles and force data, spatiotemporal parameters of the gait such as velocity, cadence, stride length, and step length are recorded. Electrical activity (EMG) for specific muscular groups is recorded using surface electrodes positioned on the subject skin. The fifth column of Table 1 lists different knee joint biomechanical gait data acquisition techniques used in the analyzed literature.

**Figure 4:**
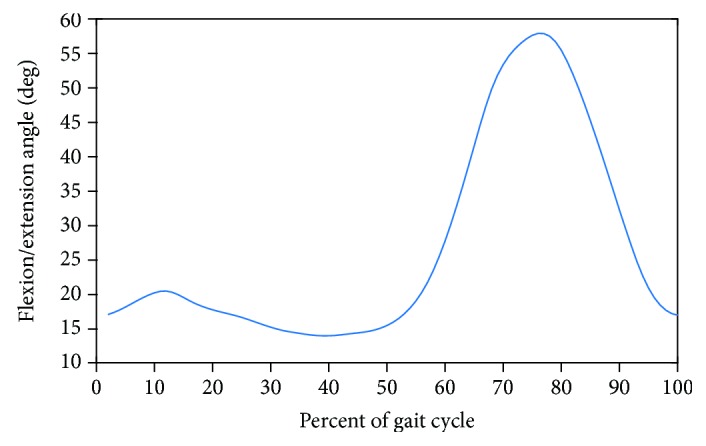
The waveform of the knee flexion angle for a normal subject is shown normalized to 100% of the gait cycle.

Spatiotemporal parameters are static numerical values, whereas kinematic, kinetic, and EMG parameters are represented as a set of time series waveforms. That is, the data are reported in two-dimensional charts, where the abscissa defines the percentage of the gait cycle (GC), i.e., the time interval from heel contact of one foot to the next heel contact of the same foot [29]. The ordinate corresponds to the biomechanical measure of interest.  Figure 4 illustrates the waveform of the knee joint flexion angle for a normal subject as normalized to 100% of the GC.

**Figure 5:**
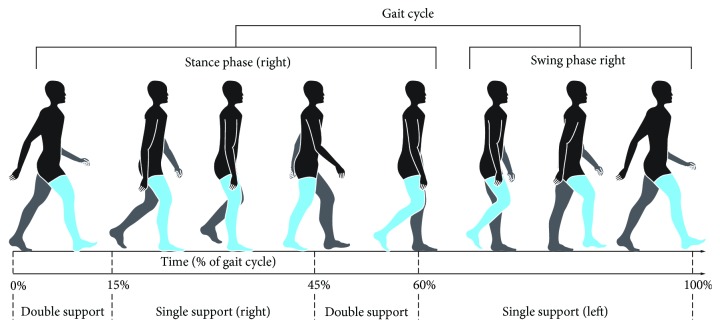
An illustration of gait phases.

The GC involves two main phases, the stance phase when the foot is in contact with the ground and the swing phase when the foot is not in contact with the ground, as illustrated in Figure 5. The stance phase generally corresponds to the first 60% of the GC, and the swing phase to the remaining last 40%. The stance phase is further composed of a period of double stance during the first and last 10% of the stance phase, when both feet contact the ground, and a period of single stance during the remainder of the stance phase when only one foot is in contact with the ground. The swing phase also has three parts: the initial swing, the mid swing, and the terminal swing.

The gait data of human motion is generally characterized by high dimensionality, time dependency, high variability, significant correlation, and nonlinearity [22].

**Figure 6:**
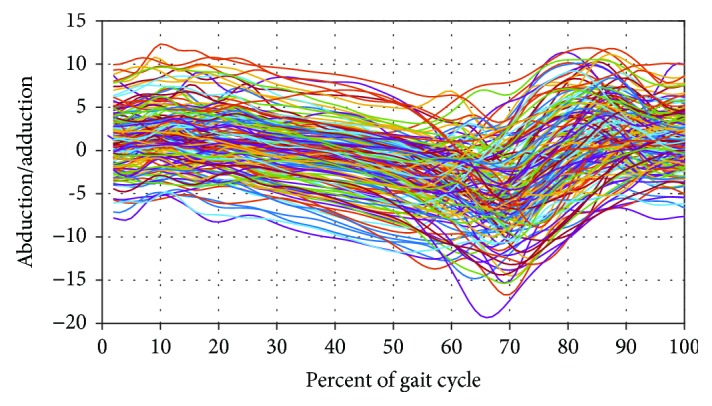
Illustration of high dimensionality and variability. The graph shows the graph of a sample of 160 distinct asymptomatic abduction/adduction waveforms, each composed of 100 measurement points.

Figure 6 illustrates both high dimensionality and variability within an asymptomatic (AS) population sample. The variability stems from either the anthropometric differences between subjects (i.e., intersubject variability), the differences in the data acquisition methodology (motion capture systems, walking speed, data processing, etc.) [30], and the presence of several different patterns in the AS gait data (i.e., intrasubject variability). Kadaba et al. reported that intrasubject repeatability was excellent for kinematic data in the sagittal plane both within a test day as well as between test days [31]. Huber et al. demonstrated that EMG signals exhibit high intersubject and intrasubject variability [32]. Repeatability of gait analysis studies performed across multiple trials, sessions, and laboratories was analyzed [33]. Deluzio et al. recognized the strong correlations between the time samples of gait waveforms [34]. Moreover, differences and similarities between gait curves were assessed [35]. Bejek et al. showed several nonlinear relationships between gait parameters [36]. After they are recorded, the time series waveforms are preprocessed to extract and select features of representation, and finally, classified.

### 3.2. Feature Extraction and Selection

**Figure 7:**
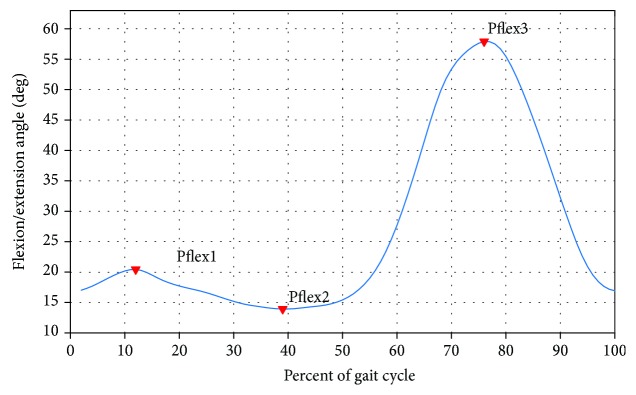
Example of representation of points of interest on kinematic waveforms.

A crucial step in knee joint biomechanical data analysis is feature extraction and selection to identify a set of informative and discriminatory features. There are two broad approaches to feature extraction: local and global. These have been described and discussed in [37]. A local method consists of describing the biomechanical data based on some specific points extracted from the biomechanical waveform. In this case, the outcomes are reported as their summary statistics (e.g., mean, variance, correlation, and range) or a parametrization (discrete variables and peak amplitudes) involving measures on a single biomechanical gait data. For example, the typical knee joint flexion/extension waveform included a stance phase peak flexion angle (Pflex1), a swing phase peak flexion angle (Pflex2), and a minimum value (Pflex3), which could be extracted as illustrated in Figure 7. Several biomechanical studies on discriminating patients with knee joint OA from the normal subjects using local approaches are available in the literature. The maximum knee flexion and abduction angles were analyzed [38]. Parameters from angle, force, moments, and acceleration, in the sagittal, frontal, and transverse planes, were used [39] to distinguish the gait of the knee with medial OA from a normal gait. In another study, five characteristics, namely, the sagittal/frontal/transverse plane range of motion along with the peak vertical GRFs and cadence, were used [40, 41]. The GRF magnitudes, the time for peak GRFs, and the subjects' velocity were measured for vertical, posterior, and anterior peaks [42]. The kinematic waveforms were characterized using 14 points of interest [37]. In [43], specific kinematic parameters, such as knee angle at initial foot contact, peak angles, minimal angles, and angle range, were extracted, which concord with those identified in the knee joint gait literature [44, 45]. In recent studies, a set of 70 features were extracted from 3D kinematic patterns based on variables routinely assessed in clinical biomechanical studies of knee OA populations, such as maximums, minimums, varus and valgus thrust, angles at initial contact, mean values, and range of motion throughout GCs or GC subphases [46–48]. Within these features, a set of 14 features have been identified as diagnostic and burden of disease biomarkers for knee OA characterization.

The local approach representation presents the ability to reduce biomechanical data into smaller meaningful features in a simple way, without compromising an accuracy rate. However, the selection process of parameters from waveforms is subject to limitations: (1) it depends highly on expert opinion that can often be contradictory and uncertain, (2) it can be time-consuming, (3) it can introduce a subjective bias in feature extraction, (4) it neglects the temporal information in biomechanical waveforms, and (5) it may omit important information contained in the full original waveform.

In contrast to local approaches, global schemes take the entire biomechanical waveform over a gait cycle into account to extract and select the features of representation. In the following, waveform methods for global feature extraction such as Singular Value Decomposition (SVD), Principal Component Analysis (PCA), and Wavelet Transform (WT) are outlined. SVD is a matrix factorization method into weighted matrices. It is used to reduce the dimension of high-dimensional data while retaining the most discriminant features, i.e., without losing information in any significant way. SVD was used to characterize the kinematic waveform while also identifying gait subcycles for a better discrimination between the AS and OA groups and for assessing the severity of the disease of OA patients into KL1-2 and KL3-4 categories according to the Kellgren and Lawrence (KL) scale [37, 49]. For AS/OA classification, the analysis showed that the most discriminant subcycle was during the stance phase. Concerning the knee joint OA severity assessment, the most discriminant subcycle was during the swing phase of the frontal kinematic waveforms and the success rate was 93.2%.

The main purpose of PCA is to summarize the most important information in the data by representing the variables in a limited number of optimal principal components (PCs). These features are optimal in the sense that they explain as much as possible of the variation present in the original variables [50]. For the discrimination of the normal and end-stage knee OA subjects, PCA was developed for waveform measures and discrete measures [51, 52]. In another study, PCA was developed for kinetic and kinematic waveforms [53]. The variables identified by the Dempster-Shafer Theory of evidence (DST) classifier [54, 55] as the best features to distinguish the OA subjects from the normal subjects are those that are often cited to be clinically relevant. For the discrimination of the Anterior Cruciate Ligament Reconstructed (ACL-R) subjects and healthy subjects, PCA was applied on kinematic waveforms [56]. The ACL-R subjects had a mean of 12 ± 2 months time from surgery and had incurred a complete ACL tear. All ACL-R subjects had a unilateral tear of their ligament, with no previous ligament injury of either knee and no history of knee surgery. Differences were found between groups in the frontal and transverse planes. Then, the PCs of the three planes were retained for classifying the status of normality using Logistic Regression (LR).

Only the frontal plane kinematics had high importance for classifying the status of normality. PCA was also used to extract meaningful pattern representative of the AS gait to separate the entire kinematic waveforms in the sagittal, transverse, and frontal planes into homogenous groups [57].

Another approach is to transform the input into a frequency domain to extract features, such as the WT domain. The WT representation allows a waveform characterization locally both in time and frequency simultaneously. To distinguish AS from knee joint OA gait patterns with a Kellgren and Lawrence (KL) [58] grade of 1, 2, 3, or 4 at the medial tibiofemoral compartment, a discriminant feature representation based on a wavelet decomposition was computed from the kinetic waveforms [59, 60]. The best discrimination rate was achieved using the anterior-posterior and the medial-lateral components of motion. In other studies, ACL-deficient patients, waiting for ligament reconstruction of the ACL, were distinguished from the AS population subjects using features computed from a discrete Daubechies wavelet decomposition of the kinematic and kinetic waveforms [61]. The abduction/adduction, tibial internal-external rotation, and the flexion-extension joint moments were identified as the most discriminant features, i.e., features that would best characterize the ACL population. A wavelet representation of kinematic data extracted in each plane separately (sagittal, frontal, and transverse planes) has been used to train a sample-encoding Kohonen network to distinguish between two types of knee OA pathologies, namely, Patellofemoral and tibiofemoral [62]. These studies confirm the benefit of using frequency domain transformations to reduce and analyze knee biomechanical gait waveforms.

Transform methods are in general objective and robust because (1) data from the entire gait cycle are considered and (2) feature extraction does not require a user intervention as the transformation is computed automatically over the whole biomechanical waveform.

### 3.3. Classification: Statistical and ML Methods

Biomechanical data classification is aimed at distinguishing automatically between the normal subjects and pathological knee patients. Two broad types of approaches can be distinguished: statistical methods and ML methods. Statistical methods have been applied to characterize usually small groups of subjects and to discover discriminant features or attributes. They typically use parametric tests such as the Student *t*-test, univariate Analysis of Variance (ANOVA), or multivariate (MANOVA and Wilkes' *λ*), as well as the Mann-Whitney *U* test. ANOVA has been used to analyze knee flexion, abduction, and rotation angle parameters during three daily activities to know whether there were statistically significant differences between normal and OA groups at different disease severity levels [38]. A Student *t*-test has been performed to investigate the differences between workers exposed to Knee Straining (KS) postures and non-KS for gait kinematic variables (peak, ranges, and minimum values) [43]. Statistical techniques are usually performed on local features, a representation that is subject to the limitations mentioned in Section 3.2. Moreover, these techniques are not readily applicable to feature combinations, to a large number of variables, or to subject rather than group classification. They can also lead to classification ambiguity due to group effects.

ML methods, rather than statistical methods, are used when larger amounts of data are available [63]. They can be divided into two broad categories: supervised and unsupervised learning methods. Unsupervised learning consists of discovering a structure in the organization of unlabelled data. Clustering is a typical unsupervised learning method. Few studies have investigated it for knee biomechanical patterns. The K-means algorithm has been used to discover clusters in EMG data, during a walking session, of the normal and ACL-injured subjects [64]. The mean and the standard deviation of each cluster were used to verify the clustering validity. The PC clustering model was applied to the frontal, sagittal, and transverse plane kinematic data [57], which led to the identification of four distinct patterns in a normal gait. The clustering quality has been verified based on the analysis of the silhouette width and with statistical evaluation by hypothesis testing. The density-based spatial clustering of applications with noise (DBSCAN) algorithm has been applied to the frontal, sagittal, and transverse plane kinematic data, which led to the identification of two representation patterns for each plane. Cluster divisions are evaluated using the silhouette index, the Dunn index, and connectivity [65].

Classification by supervised learning methods uses labelled data, rather than unlabelled as with unsupervised learning schemes. We focus here on knee biomechanical gait classification. Current supervised classifiers can be divided into four types.

#### 3.3.1. Tree-Based Classifiers

Tree-based classifiers are common, mainly because they are easy to interpret and implement. Five studies investigated tree-based classification. Regression Trees (RTs) have been applied to feature-based OA (with predominantly medial compartment knee OA) vs. non-OA discrimination and to grade OA severity (according to the KL grades 1 to 4) [47, 48]. The success rate of the RT classifier was 86% to distinguish KL1-2 from KL3-4 grades, 88.2% for KL1 from KL2 grades, and 88% for KL3 from KL4 grades. A regression model, the Classification and Regression Tree (CART), was used to classify patients with knee OA (bilateral OA, left knee OA, and right knee OA) according to disease severity (OA grades 1-4) using spatiotemporal gait analysis [66, 67]. Spatiotemporal parameters include velocity, cadence, step and stride lengths, base of support (BOS), step time, swing time, stance time, single limb support (SLS) time, and double limb support (DLS) time. The accuracy of the classification was 90.8% for males and 89.5% for females. All misclassifications were off by a margin of error of 1, e.g., grade 1 can be misclassified as grade 2, but never as grade 3 or 4. The most differentiating variables for classification are stride length and cadence. Using features extracted from the waveforms, another study investigated CART to classify surgical versus nonsurgical patients with a primary diagnosis of moderate to severe knee OA and scheduled for arthroplasty consult [46].

#### 3.3.2. Support Vector Machine (SVM)

An SVM is a discriminative classifier formally defined by a separating hyperplane. SVMs have been initially developed for binary classification. The multiclass SVM is an extension of the binary SVM to more than two classes. Using a multiclass SVM on vertical and anterior-posterior GRFs, Levinger et al. classified patients with different knee pathologies: Patellofemoral Pain Syndrome (PFPS), knee OA, and patients after Total Knee Replacement (TKR) [42]. A follow-up investigation applied SVMs to distinguish between the spatiotemporal gait parameters (walking velocity, cadence, stride length, stride time, step time, step length, single support time, and double support time) of OA patients who had undergone unilateral knee replacement surgery and healthy controls [68, 69]. Only two features have sufficient discriminative power to accurately classify the two groups. A Decision Tree- (DT-) based multiclass SVM has been applied to separate AS and OA patients and assess OA severity according to the KL scale by employing GRF measurements [70]. Results show that class (AS) of asymptotic gait (healthy) signals is almost perfectly separable, achieving the testing percentage of 97%. The majority of errors occur in moderate (OA) and severe (OA) arthritis categories. In particular, five moderate OA gaits are misclassified as severe OA and one as AS leading to 89.09% testing rate. Further, five severe OA gait signals are classified as moderate OA, which corresponds to a performance of 91.52%. An SVM was also trained to distinguish kinematics of patients with an ACL-injured knee from the healthy subjects [71]. ACL patients had either a knee extension or flexion deficit or a combination of both in the affected limb, but were able to walk without a walking aid for a minimum of 10 m, and sustained a complete unilateral ACL rupture within a period of 21 days (13 (SD 5) days) prior to the experiment.

#### 3.3.3. The Bayes Classifier

Two studies applied a Bayes classifier on PCs of GRFs to distinguish the knee OA subjects (OA can affect the medial or lateral tibiofemoral compartment or the patellofemoral or combination of these) from the healthy subjects [72] and to determine if workers exposed to KS have knee kinematic data that resemble those of knee OA patients rather than of non-KS workers on the first 20-GC percentages of the kinematic waveforms [73].

#### 3.3.4. Artificial Neural Network (ANN)

One study applied ANN to classify knee joint biomechanical gait data. This study trained a Multilayer Perceptron (MLP) with kinetic, kinematic, and spatiotemporal features (walking velocity, single support, and step length) to distinguish the healthy subjects from knee OA subjects [74]. It included two experiments and reported the accuracy rates. In the first experiment, the data set was partitioned into five subsets and five MLPs were correspondingly trained and tested. Then, combination rules produced the final classification. In the second experiment, the entire data set is used to train an MLP.

## 4. Discussion and Conclusions

Reliable diagnosis of knee joint pathologies can be a complex task, requiring in many cases a combination of roentgenographic data (magnetic resonance imaging and computed tomography) and clinical tests. This complexity mirrors that of the joint and its six degrees of freedom motion. The literature review shows that *pattern classification of knee joint biomechanical data can assist diagnosis* and, therefore, lessen the burden of this complexity.

A few studies have addressed knee joint pathology classification based on knee biomechanical data. We note that the subject is of recent interest in research because all of the relevant literature dates from 2000 up (second column of Table 1). The 31 studies retained for the literature review have in common the methodology adopted for the classification of normal and pathological knee function.

### 4.1. Biomechanical Data Acquisition

Biomechanical gait data are collected using a gait measurement setup usually composed of force plates, a set of markers fixed on an attachment device, and a motion capture system. The acquired data consists of spatiotemporal parameters, kinematics and kinetic measurements, and EMG (in the form of time-dependent functions (time series) with the abscissa as percentage of the GC and the ordinate as the gait measurement of interest). Most of the reviewed studies have used either spatiotemporal gait analysis, kinematic, kinetic, or combination of those features to be fed into machine classifiers to distinguish between individuals with and without knee pathology. There is a wide variety of biomechanical data acquisition systems from different laboratories, some listed in column 5 of Table 1. However, the heterogeneity of these biomechanical data acquisition systems hinders interoperability and sharing of biomechanical gait data among collaborative laboratories. Most of the reviewed studies have concentrated on patients having OA because it is the most common disease affecting the knee joint [75]. Spatiotemporal parameters, which span the time and distance dimensions, are more descriptive and easier to interpret clinically [76] and, as such, have been used to differentiate between the healthy individuals and patients with pathological knees. In particular, the single limb support (SLS) was shown to be a good discriminatory indicator for OA [77, 78]. However, spatiotemporal parameters do not include measurements of joint motion and can miss important information as a result. Moreover, such parameters are not specific enough to reliably detect the subtle biomechanical differences involved in knee OA [79]. It has also been demonstrated that spatiotemporal variables are not good parameters to differentiate knee gait biomechanics of the ACL-R subjects from the healthy ones and should not be used as criteria to determine the return to sports after ACL-R, since the ACL-R group did not show differences in spatiotemporal gait parameters related to a control group [80]. Consequently, gait investigations for knee OA understanding were mostly based on three-dimensional kinetic and kinematic patterns. Traditionally, kinetics, particularly the external knee adduction moment, has been used to assess the progression and severity of knee OA. However, their measurement needs sophisticated setups, which are, generally, available only in specialized gait study laboratories. One of the instruments used to analyze the kinetics of different human body joints is the force plate or force platform. However, force plates are usually expensive and appear to be more suited to research than to the clinical environment [81]. Kinematic data, instead, are acquired in a normal clinical setting, using generally a commercially available treadmill and a simple noninvasive knee attachment system. Hence, in order to facilitate the use of biomechanical evaluations in the clinical environment, biomechanical investigations of the knee were limited in most of the reviewed studies to the kinematic parameters.

### 4.2. Feature Extraction and Selection

The most serious impediments for the clinical application of knee biomechanical gait data are the high data dimension and the significant data variability. Variability stems from differences in data collection methodologies and the presence of several different biomechanical patterns inherent to individuals. The high variability of biomechanical gait data and the curse of dimensionality have constrained most studies to apply directly traditional analytics. Further difficulties may stem from the need for an expert interpretation. Therefore, it is crucial to develop more efficient, automatic, and objective techniques for dimensionality reduction. Current studies agree that appropriate data representations of biomechanical data and pattern recognition paradigms can overcome the aforementioned difficulties to produce reliable systems which can classify knee pathologies. The literature review shows that data dimensionality reduction is useful. It is aimed at simplifying the biomechanical data without loss of information for classification. Both local and global features can serve dimensionality reduction. Local features most often considered are characteristic points on the data waveforms, such as peaks. Being sensitive to the high variability of knee biomechanical patterns, characteristic point selection can be subjective and rely on human expert knowledge, elicited from clinical professionals for instance. It can also overlook meaningful information since their definition relies only on local temporal neighborhoods. Even though local feature representation may be simpler for clinicians to understand and interpret, the most widely and readily applicable is the global features, which consider the entire GC. Both transforming the data to a frequency domain, as with wavelet decompositions, and principal component analysis (PCA) representations have often been successful. PCA methods allow the results to be interpreted by visualization, which is quite convenient. The features elicited by PCA often agree with the most clinically relevant features, which is a strong vote for the scheme. The literature review reveals that in spite of the relevance of domain transform and PCA representations of data, the resulting feature vector potency largely depends on the input data. One popular approach to feature selection consists of using particular classifiers, the NNC, for instance, to classify the features from which to select a subset, by cross validation on test-and-training divisions of data, and where the most performing features are retained. Of course, although this method provides the best performing discriminatory features for the specific classifier used, there is no guarantee that this selected set will perform best with other classifiers.

### 4.3. Classification

There are currently several classification methods of knee biomechanical data, namely, ANNs, SVMs, DTs, and the Bayesian classifier. These work well with multidimensional data. Also, they can be used to extract important pathology-related features when combined with feature extraction, hence assisting diagnosis for more accuracy. The most often used supervised learning classifier is the SVM. By contrast, investigations with ANN are scarce. When the target classes are unavailable, i.e., with unlabeled data, clustering is the means of classification. These classifiers have been successful in identifying groups from biomechanical data and able to rank features by power of discrimination. Very large volumes of data are generally acquired for analysis in any given biomechanical study, in some cases to the point where one can consider treating what is often called a “big data” problem [82]. However, most studies in the field of knee biomechanics generally involve only a few variables and low subject numbers (e.g., 5-30 subjects). So, it is questionable whether these methods are efficient in this case, since generalization is the central challenge in ML. Although most of the studies continue to involve only a small cohort of subjects in the analysis, a larger cohort of the subject could improve the classifier's performance. Generalization capabilities of the classification scheme have not been confirmed. Moreover, classification involved recognizing pathological biomechanical patterns of the knee from the healthy and control subjects. In other words, biomechanical studies assume that all subjects to be classified belong to exactly one of the two well-defined classes (pathological or AS). However, the vast majority of normal human actions do not belong to the well-defined classes. It may not be accurate enough to classify an individual as AS or pathological, and a refined diagnosis may be a requirement. Moreover, it is not clear from these studies that the same technique would be useful for discriminating patients with other pathologies or patients with more than one pathology. All of these factors explain in large part why clinicians have not yet fully adopted the analysis of biomechanical data as a diagnostic aid.

### 4.4. Performance Measures

It is essential to compare systematically and thoroughly the performance of the ML algorithms currently in use, given their potential for biomechanical data classification for knee pathology diagnosis. Accuracy metrics were extracted from the reviewed studies, when supervised learning techniques are used. However, it is difficult to provide a like-by-like comparison between these studies, due to the lack of interoperability between the often different acquisition systems used in different laboratories, as explained in Section 4.1. Execution time and computational complexity have not been computed in the reviewed articles.

### 4.5. Future Work

The studies mentioned in this review may serve as guides for stating and solving a complex classification problem, where we can classify knee biomechanical data into different disease-related classes with a larger number of training samples. The use of a computer-aided system based on ML techniques, specifically ANNs, is a promising prospect in the field of knee biomechanical data classification. The basic need is to design the right set of features for knee biomechanical data classification and then provide these features to a classifier. However, the feature representation that provides optimal classification performance is still an open issue. One solution may be deep learning which is motivated by the failure of traditional algorithms to generalize well on knee joint biomechanical data classification task. Deep neural networks have shown a great performance in image, text, and audio classification problems compared to conventional methods.

The advantage of deep neural networks is that they have an automatic feature extraction component from raw, complex, and high-dimensional data. Learned representations often result in a much better performance than can be obtained with hand-crafted representations. Moreover, the resulting feature vectors are generic and transferable. As a result, deep neural networks generalize well to new input data. We hypothesize that the use of deep neural networks will be quite useful for the automatic classification of knee kinematic data.

## Figures and Tables

**Table 1:** Knee joint biomechanical gait data classification-related studies.

Study	Pathology	Population	Biomechanical variables	Data acquisition	Feature Ext/select	Classification	Acc
[64]	ACL	20 ACL 26 injured knees	EMG	PDP 11/23 minicomputer	Fourier transform	K-means	—
[51]	OA	50 OA 63 AS	Spatiotemporal data Kinematics Kinetics	Optoelectronic measurement system Force plate	Global rep. *PCA*	LDA	94%
[38]	OA	35 OA 107 AS	Kinematics	3D knee analyzer	Local rep.	ANOVA	—
[39]	OA	12 OA 7 AS	Acceleration data Kinematics Kinetics	Vicon motion analysis system Force sensor integrated treadmill triaxial gyroscopes and accelerometers	Local rep.	LDA	87.9%
[40]	OA	15 OA 15 AS	Spatiotemporal data Kinematics Kinetics	Optoelectronic measurement system Force plate	Local rep.	DST	96.7%
[41]	OA	20 OA 25 AS	Spatiotemporal data Kinematics Kinetics	Optoelectronic measurement system Force plate	Local rep.	DST	—
[74]	OA	110OA 91 AS	Spatiotemporal data Kinematics Kinetics	Vicon motion analysis system	—	MLP	98.5%
[52]	OA	50OA 63AS	Kinematics Kinetics	Optoelectronic measurement system Force plate	Global rep. *PCA*	LDA	92%
[42]	PFPS-OA	13PFPS 5OA 20 PKR	Kinetics	Kistler force platform	Local rep. Hill-climbing algorithm	SVM	85-92%
[69]	OA	11 OA 12 AS	Spatiotemporal data	Vicon motion analysis system GAITRite system	Hill-climbing algorithm	SVM	94.2%
[53]	OA	20 OA 22 AS	Kinematics Kinetics	Optoelectronic measurement system Force plate	Global rep. *PCA*	DST	97.62%
[60]	OA	26OA 16AS	Kinetics	Kistler force platforms	Global representation *WT*	NN	91%
[59]	OA	26 OA 16 AS	Kinetics	Kistler force platforms integrated to an ADAL treadmill	Global rep. *WT*	NNC	90%
[68]	OA	11 OA 12 AS	Spatiotemporal data	Vicon motion analysis system GAITRite system	Hill-climbing algorithm	SVM	88.89%
[70]	OA	24 OA 12 AS	Kinetics	Bertec platform	Wavelet packet based on the Fuz-Coc	DT-SVM	93.44%
[37]	OA	30 OA 14 AS	Kinematics	KneeKG	Local rep. Global rep.	SVD	77.27% 93.18%
[61]	ACL	29 ACL 15 AS	Kinematics Kinetics	ADAL treadmill Vicon motion analysis system	Global rep. *WT*	NNC	83.2%
[49]	OA	30 OA 14 AS	Kinematics	KneeKG	Global rep.	SVD	93.1%
[56]	ACL-R	6 ACL-R 10 AS	Kinematics	Four cameras motion analysis system (Innovision)	Global rep. *PCA*	LR	93.75%
[43]	OA	18 KS 20 non-KS	Kinematics	KneeKG	Local rep.	Student's *t*-test	—
[57]	AS	111 AS	Kinematics	KneeKG	Global rep. *PCA*	Discriminant model based on PCs' sign	—
[66]	OA	2,900 OA	Spatiotemporal data	Computerized walking mat	—	CART	89.5%–90.8%
[67]	OA	2,911 OA	Spatiotemporal data	Computerized walking mat	—	CART	89.5%–90.8%
[72]	OA	47 OA 133 AS	Kinetics	Kistler force plates	Probabilistic PCA	Bayes classifier	82.62%
[73]	OA	25 KS 25 non-KS 29 OA	Kinematics	KneeKG	—	Bayes classifier	—
[46]	OA	44 S 40 non-S	Kinematics	KneeKG	Local rep.	CART	84.7%
[48]	OA	100 OA	Kinematics	KneeKG	Local rep.	RTs	88%
[71]	ACL	7 ACL 7 AS	Kinematics	Vicon motion analysis system	PCA	SVM	100%
[47]	OA	100 OA 40 AS	Kinematics	KneeKG	Local rep.	RTs	85%
[65]	AS	165 AS	Kinematics	KneeKG	Isometric mapping	DBSCAN algorithm	—
[62]	OA	63 OA	Kinematics	KneeKG	WT	Kohonen neural network	90.47%

## Abbreviations

EMG: Electromyography
GRFs: Ground Reaction Forces
OA: Osteoarthritis
ML: Machine Learning
3D: Three-dimensional
GC: Gait cycle
AS: Asymptomatic
SVD: Singular Value Decomposition
PCA: Principal Component Analysis
WT: Wavelet Transform
PCs: Principal components
DST: Dempster-Shafer Theory of evidence
ACL: Anterior Cruciate Ligament
LR: Logistic Regression
ANOVA: Analysis of Variance
RTs: Regression Trees
CART: Classification and Regression Tree
SVM: Support Vector Machine
PFPS: Patellofemoral Pain Syndrome
TKR: Total Knee Replacement
DT: Decision Tree
ANN: Artificial Neural Network
MLP: Multilayer Perceptron
PNN: Probabilistic Neural Network
LDA: Linear Discriminant Analysis
S: Surgical
NNC: Nearest Neighbor Classifier
KS: Knee Straining
PKR: Post Knee Replacement.
